# “O Sister, Where Art Thou?”—A Review on Rescue of Imperiled Individuals in Ants

**DOI:** 10.3390/biology10111079

**Published:** 2021-10-22

**Authors:** Krzysztof Miler, Filip Turza

**Affiliations:** 1 Institute of Systematics and Evolution of Animals, Polish Academy of Sciences, 31-016 Kraków, Poland; 2 Institute of Environmental Sciences, Faculty of Biology, Jagiellonian University, 30-387 Kraków, Poland

**Keywords:** ants, altruism, cooperation, empathy, Formicidae, helping, rats, pro-social behavior, rescue behavior, risky behavior

## Abstract

**Simple Summary:**

Ants provide an outstanding example of organisms capable of risky acts. When ants engage in rescue behavior, for example, they do so for a chance of saving another individual from a dangerous situation. What contributes to whether a particular ant engages in rescue behavior? Why do some species of ants show high rescue activity while other species show no such behavior at all? How is rescue behavior triggered in ants? Finally, but no less importantly, how risky engaging in a rescue action really is and what benefits it brings to both the rescuing and rescued ant? These are the fundamental questions we address here. We demonstrate the progress in the research field and, in doing so, we expose the extent to which the abovementioned questions are unanswered. In this comprehensive review, we present a summary of relevant published works and hope to spin higher interest in the fascinating area of study that is ant rescue behavior.

**Abstract:**

Altruism is defined as an action that decreases the lifetime direct fitness of an actor and benefits one or more recipients. This phenomenon, which is generally difficult to understand and explain, requires special research attention. The subject of this review, rescue, is a type of altruistic behavior in which the actor puts itself at risk to save another individual, the recipient, that is in danger. The highest numbers of published empirical works have been devoted to rescue behavior in ants and they have enormous potential for further study. We review studies devoted to the subject and group them into four main areas of research on ant rescue actions: (1) variation in rescue behavior activity on a between-individual scale, (2) factors contributing to the evolution of rescue behavior on a between-species scale, (3) rescue behavior releaser signals and (4) rescue behavior benefits and costs. We highlight the progress in research on rescue behavior in ants, indicate that this behavior is probably much more common than previously thought yet thus far demonstrated in only a few species, and uncover research gaps and open questions that remain unexplored. We additionally point out some gaps in knowledge that become evident when research devoted to rescue behavior in rats, the second most studied group of animals in this context, is briefly overviewed. We hope to help navigate among studies on rescue behavior and provide the most up-to-date summary of the relevant literature. Moreover, we hope to encourage and facilitate researchers in behavioral ecology and other subdisciplines to further experimentally analyze rescue behavior, not only in ants but also in other taxa.

## 1. Introduction

Cooperation is a type of interaction in which at least two organisms act together for their shared benefit and is often demonstrated by social animals [1]. The highest level of social organization among animals, eusociality, exemplifies large-scale and extraordinarily effective cooperative behavior [2]. Cooperation among eusocial insects, such as ants, emerges in many behaviors including, for example, brood care and anti-predator defenses. Ants’ cooperation brings them benefits that maximize fitness and, thus, is considered one of the main reasons for their ecological dominance in many habitats [3]. Indeed, some authors suggest that eusociality, represented by ant societies, evolved precisely as multigenerational “life insurance” in which group-living, highly related and cooperating individuals may carry on various investments of their predecessors, multiply them and have them completed by their successors [4]. There are several prominent, attention-grabbing areas of highly cooperative behavior of ants, such as hygiene [5], decision-making [6] and transport [7]. Complex cooperative behavior in ants often serves as an inspiration for solving mathematical problems and for explaining phenomena essential for the survival of societies. For example, ant algorithms developed based on how real ants find the shortest paths between food sources and their nest provide solutions to such optimization problems as the travelling salesmen problem or the vehicle routing problem [8]. Similarly, the adaptive plasticity of social networks in ants exposed to an epidemic risk suggests effective ways for mitigating the effects of pathogens in social groups [9].

There is another, seemingly grossly different from cooperation type of interaction between organisms, namely altruism. Altruistic action, on average, decreases the lifetime direct fitness of an actor and benefits one or more recipients [10]. Striking examples of altruism span the animal kingdom and include, for example, matriphagous spiders [11]. The consumption of the mother by her offspring allows for body mass gain and enhanced molting time in the offspring, but at the same time causes loss of life in the mother. The persistence of altruistic actions, although somewhat puzzling, stems from their ultimate benefit to the actor, obtained indirectly through, for example, kin selection [10]. In the mentioned spiders, the mother may desert her clutch shortly before matriphagy and produce more offspring later. However, reproductive output for the mother is likely greater when she allows the first clutch to eat her than when she escapes cannibalism and produces another clutch [11]. In eusocial insects, including ants, an example of altruistic action is provided by workers abandoning their reproduction in favor of rearing their (usually very close) colony relatives, the queen’s offspring [12]. While this kind of reproductive altruism is at least partially coerced [13], it persists largely due to the indirect benefits it brings to each individual actor. Eusocial insects also show more “voluntary” altruistic behaviors for which there is no evidence of coercion. Self-destructive defensive behavior illustrates this well in a number of eusocial taxa, ants included [14]. For example, some *Forelius pusillus* individuals close the entrance to their colony nest from the outside, sealing their own fate by exposing themselves to predators and exhaustion [15]. Similarly, sick *Temnothorax unifasciatus* ants leave their nest to die alone, thereby preventing disease spread in the colony [16]. Another altruistic behavior present in ants, but not connected to defensive self-sacrifice, is the subject of this review.

This behavior, rescue, is an interaction between one individual, the rescuer, and another individual, the victim. Rescue behavior involves four components [17,18]: (1) the victim is endangered and at immediate physical risk, (2) the behavior of the rescuer is suited to the circumstances of the victim’s endangerment, (3) the rescuer places itself at risk by engaging in rescue behavior, and (4) the act of rescuing is not inherently rewarding or beneficial to the rescuer. Regarding the first component, endangerment might be more or less direct, but it must involve a risk of severe fitness loss. For instance, if the victim is not rescued, it will be preyed on by a predator. The second component, in turn, specifies that what the rescuer does is not random or accidental but functions to lower (or eliminate) the victim’s endangerment. For example, an individual approaching the victim only to contact it and then walking past it without providing help is not rescuing. This is more detailed by the third component because what the rescuer does must incur some level of direct cost. Such a cost might include, for instance, energy expenditure, or the risk of becoming another victim. Finally, the fourth component specifies that the behavior of the rescuer is only indirectly advantageous to it. As such, it leads to no benefits in terms of the rescuer’s direct fitness, particularly reproduction or survival.

### Aim of the Review

Below, we present a detailed overview of the published works on rescue behavior in ants, with the purpose of providing an up-to-date summary of research on the subject. We also provide a broader perspective by briefly discussing studies devoted to rescue behavior in rats. In doing so, we identify gaps in knowledge that need further experimental attention. The review is, in our opinion, timely, because (1) there is no other such comprehensive review on the subject, with earlier summaries discussing only selected aspects of the issue [18,19,20]; (2) new findings, especially those regarding determinants of the expression of rescue and factors contributing to the evolution of rescue, accumulated and need to be underlined; (3) highlighting areas of insufficient support and integrating valid conclusions from relevant studies are currently lacking; and (4) new and greater interest in the subject is desirable, which is something we hope to elicit with this review.

## 2. Overview of Works on Rescue Behavior in Ants

A starting point for studies devoted to rescue behavior in ants was provided by Belt [21], Wheeler [22] and Lafleur [23], who reported anecdotal observations of ants stuck under stones, on the surface of water, and trapped in clay as being helped by nestmates. These observations were made in the 19th and in the first half of the 20th century. 

A first more detailed study was provided by Markl [24] in the leaf-cutting ant *Atta cephalotes*. The author noted that these ants, when buried under an earth slide, produced stridulatory signals audible even to humans. When artificially trapped in glass vials buried partially in the ground in proximity to the nest, ants elicited certain reactions in their nestmates. First, nestmates were attracted to individuals stridulating inside vials, and second, they displayed digging behavior and attempted to reach the trapped ones. Similar observations were made by Spangler [25] in *Pogonomyrmex occidentalis* ants and by Hangartner [26] in *Solenopsis geminata* ants. However, in the case of these two species, it was not stridulation that elicited the reactions, but chemical signals. In the case of *P. occidentalis* these signals were unknown pheromones, probably originating from the mandibular glands, while in the case of *S. geminata* they constituted primarily CO_2_ released by the body. 

**Figure 1:**
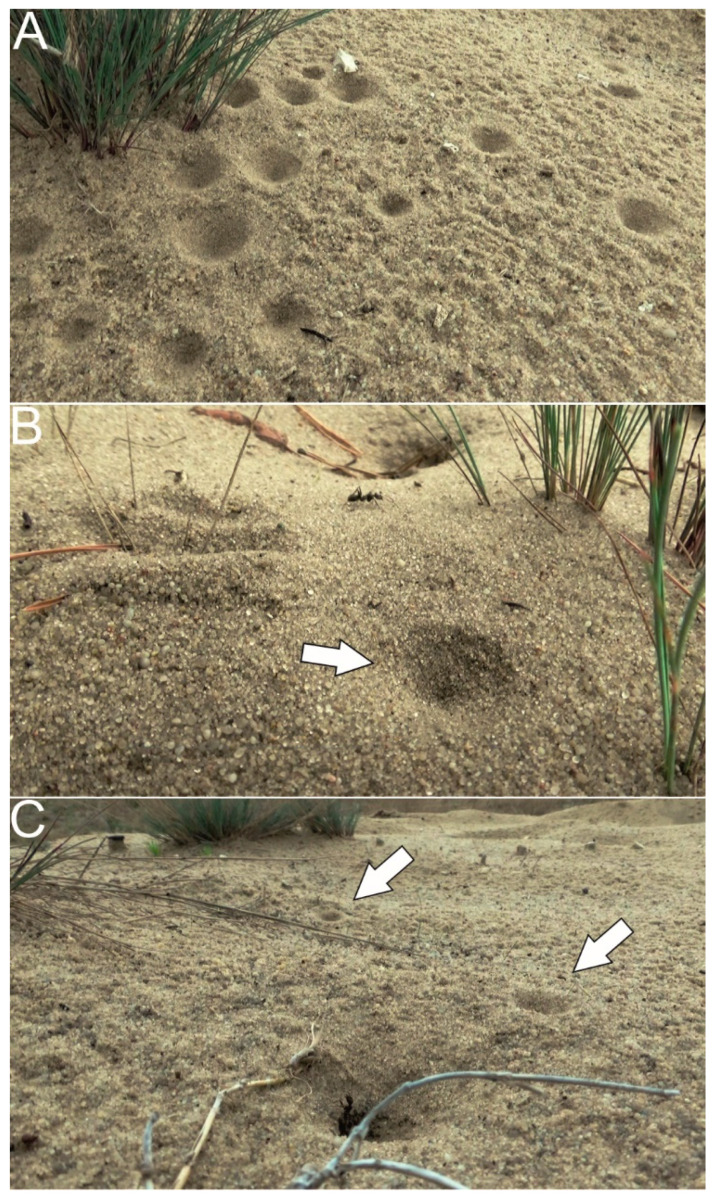
Ant rescue actions in the field might be observed in the context of capture by predatory antlion larvae. Aggregation of traps built by antlions in the habitat shared with sand-dwelling ants, such as *F. cinerea* (**A**). Traps of antlions (indicated by arrows) close to nest entrances of *F. cinerea*, with visible forager workers (**B**,**C**). The traps are 2–5 cm in diameter. Photographs: F. Turza, Błędowska Desert (southern Poland).

Only much later, in 2002, Czechowski et al. [27] provided observations of “rescue actions” in ants in another life-threatening situation—capture by predatory antlion larvae. Antlion larvae, many species of which inhabit sandy areas and build pitfall traps, hunt for small arthropods, mainly ants (Figure 1) [28]. The authors found that the sand-dwelling *Formica sanguinea* and *F. cinerea* ants, when caught by antlions, elicited vigorous “rescue behavior” in their respective nearby nestmates. The rescue behavior took the form of pulling at the captured individuals and digging in the sand surrounding them. In contrast, identical situations elicited no rescue behavior in another sand-dwelling ant species, *F. fusca*, when nestmates were captured. The authors, in light of the studies mentioned earlier [24,25,26] as well as some other studies, focused more on the identification of chemical substances that elicited alarm and digging behavior in various species of ants [29,30,31], discussed possible rescue behavior releaser signals in the studied species. These signals potentially included compounds present in mandibular, anal or venom gland secretions. Rescue behavior in ants in the context of capture by predatory antlions seemed truly risky and potentially costly.

**Figure 2:**
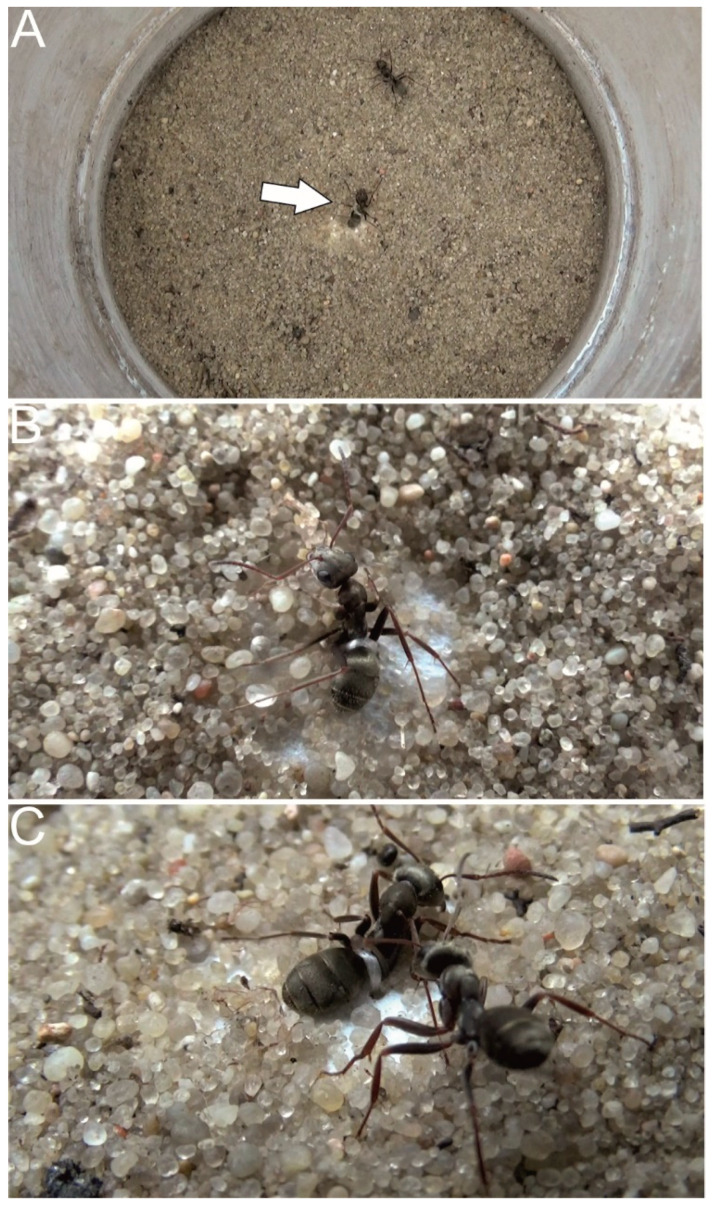
Artificial nylon snare situation arranged for *F. cinerea* workers based on the design proposed by Nowbahari et al. [32]. Test arena with the victim ant (indicated by an arrow) immobilized using a nylon snare and the potential rescuer ant, which is a nestmate of the former (**A**). Close-up view of the victim, with a visible nylon thread tying it to the piece of paper hidden underneath the sand surface (**B**). Rescue action, with the rescuer pulling at the leg of the victim (**C**). Photographs: F. Turza.

Experimental study of rescue behavior in ants began in 2009, with a paper by Nowbahari et al. [32]. The authors tested the hypothesis that kinship between the receiver of rescue and the giver of rescue determines rescue expression. The authors addressed this question by investigating the response of the sand-dwelling *Cataglyphis cursor* ants, in which they observed some rescue actions in the field, to endangered homocolonial (i.e., individuals of the same species from the same colony), heterocolonial (i.e., individuals of the same species from another colony) and heterospecific (i.e., individuals of another species) ants. For that purpose, they developed a laboratory “artificial nylon snare situation”—a simulation of an ant becoming entrapped by collapsing sand or debris that, in ants such as *C. cursor*, was a plausible ecological situation in which these ants might find themselves in need of rescue. In the simulation, the “victim” individual was tied using a nylon thread to a piece of paper and placed on an arena, where it was partially buried beneath the sand surface (Figure 2). By manipulating the “potential rescuers” introduced into the arena—five individuals from the same colony, from another colony or from another species—they were able to measure rescue activity under varying conditions of relatedness between the victim and the potential rescuers. The authors found that rescue actions occurred exclusively in the homocolonial condition. Rescue behavior was expressed as sand digging, sand transport, pulling at the legs of the victims and, remarkably, biting the nylon snares. That last behavioral category was not a response to a foreign object because nylon threads, when not used for entrapping homocolonial victims, were met with total indifference from the ants. Furthermore, because the authors included a control group of anesthetized victims (i.e., an additional test type with the victim in a chill coma and five homocolonial potential rescuers), they were able to conclude that rescue behavior releaser signals, in the form of some pheromones, were emitted only by active victims. This was because they observed no rescue attempts towards anesthetized victims.

In another study by Nowbahari et al. [33], the authors investigated the effect of temporal polyethism on the expression of rescue behavior in the same *C. cursor* ants. The authors leveraged the fact that in these ants, as in many other species, individuals progress in the type of tasks they engage in as they age. In *C. cursor*, the youngest “inactives” do little besides maturation; then, they transition into “nurses” specializing in brood care and, finally, become “foragers”, which find and collect resources in the field. The authors, using the same laboratory simulation of entrapment as described above, performed tests with forager, nurse or inactive victims in interaction with five foragers, nurses or inactives as potential rescuers (i.e., full-factorial design). They found that inactives elicited almost no rescue behavior compared to nurses and foragers, regardless of the type of potential rescuers. Furthermore, they found that foragers displayed the highest levels of rescue behavior performance, with the lowest levels in inactives and intermediate levels in nurses. The authors also reported that inactives never showed any biting of the snares, which was relatively frequent in foragers and intermediate in nurses. These results were interpreted in the context of the physiological maturation of individuals, primarily their glandular development. Their hypothetical ability to “call for help” and their sensitivity to rescue-eliciting calls of other individuals were said to be lowest in inactives, highest in foragers and intermediate in nurses.

The first comparison between the rescue behavior demonstrated by different ant species was provided by Hollis and Nowbahari [34]. The authors performed tests by ensnaring the victims as in the laboratory simulations of entrapment, but instead of placing them in a controlled arena, they placed them in proximity to the nest entrance in the field. It was the first experiment on rescue behavior performed in field conditions. The authors used *C. floricola*, *Lasius grandis*, *Aphaenogaster senilis*, *Messor barbarus* and *M. marocanus* ants as their study subjects and found high levels of rescue behavior in the first two species but negligible levels in the remaining ones. These results were discussed in the context of the ecological characteristics of the species under study. Specifically, the authors argued that the sand-dwelling ants (*C. floricola* and *L. grandis*, similar to *C. cursor*), all of which display rescue and are characterized by solitary foraging activity and nesting in the sand, can potentially meet with antlion predation and situations such as becoming trapped under collapsed sand. In turn, *Messor* ants, both of which fail to display rescue and are characterized by supposedly safer foraging in trails and nesting in compact soils, might have no real need for rescue behavior, which is why they show no signs of it. The data seemed to fit this ecological pattern nicely, although increasing the number of studied species was highly desirable for more definitive results. This was all the more so considering that *A. senilis*, another sand-dwelling species tested by the authors, was a clear exception from the pattern and showed no rescue behavior.

These results were expanded by Taylor et al. [35]. Using a similar setup as Hollis and Nowbahari [34], the authors demonstrated that rescue behavior occurred in *Tetramorium* sp. E, but not in *Prenolepis imparis* ants. The two studied species, however, shared similar sandy habitats in which they co-occurred with predatory antlions. Notably, in this context, in the study of Czechowski et al. [27], although the number of observations was low, rescue behavior was never observed in the sand-dwelling *F. fusca* ants—in contrast to *F. sanguinea* and *F. cinerea* inhabiting the same areas. Earlier studies also provided clues that ecological factors contributing to rescue behavior occurrence in different species go beyond the type of foraging activity and nesting habitat [24,25,26]. Therefore, the proposition that these factors determine whether a given species demonstrates rescue behavior or not holds only partially and has several exceptions. Interestingly, Czechowski et al. [27] initially proposed that the presence or absence of rescue behavior in a species might depend on its position in the interspecific competition hierarchy [36], which is based on the social organization of species: species that defend only their nest (group 1), species that defend their nest and food sources (group 2), and territorial species that also defend their whole foraging area (group 3). Therefore, the authors proposed rescue actions to be most likely in species belonging to group 3, characterized by the highest levels of cooperation outside the nest, which was in line with their observations in *Formica*: rescuing *F. sanguinea* and *F. cinerea* belonged to group 3, and non-rescuing *F. fusca* belonged to group 1. However, later published studies (e.g., [32,33]) cast doubt also on this idea. Indeed, *Cataglyphis* ants lack territoriality [37] but demonstrate very high levels of rescue behavior. Thus, it became clear that unconsidered ecological factors might be involved in the issue of rescue behavior presence or absence in different species. In some cases, a lack of rescue activity in a species might stem not from it being unnecessary, but from various reasons constraining the evolution of appropriate behavior.

**Figure 3:**
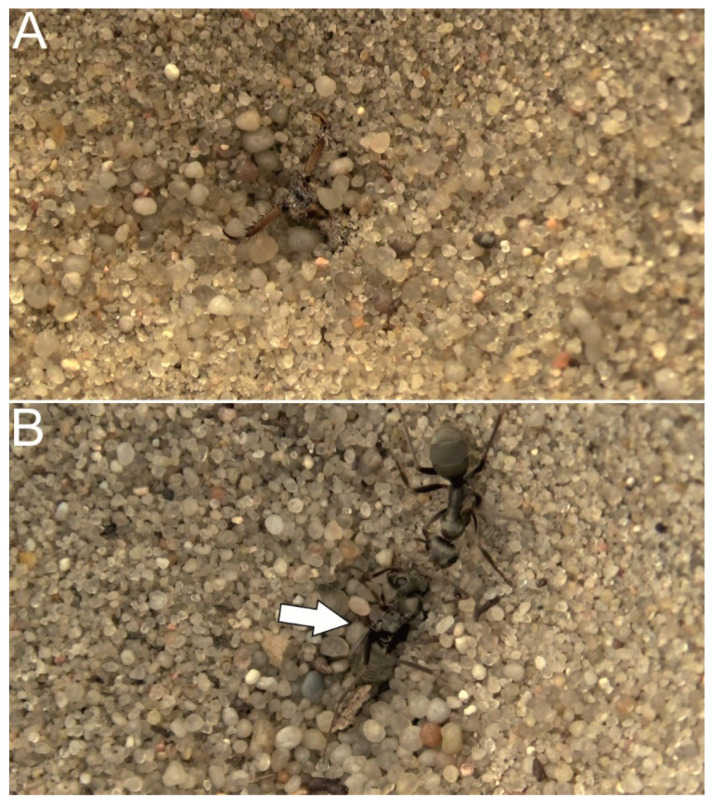
Rescue behavior test with the use of a predatory antlion larva. Close-up view of the antlion in wait for the prey at the bottom of the funnel-shaped trap (**A**). The victim ant (*F. cinerea*), captured by the antlion, and the potential rescuer (nestmate of the victim), making contact (**B**). The arrow indicates the mandibles of the antlion, holding the victim in place. Photographs: F. Turza.

The abovementioned study of Taylor et al. [35] provided two other valuable results. First, the investigated *Tetramorium* sp. E ants were tested with endangered homocolonial and heterocolonial victims. Interestingly, however, unlike *C. cursor* [32], these ants build extensive interconnected nests between which queens and workers move without aggression. Therefore, the authors predicted rescue to occur in this species in response to both homocolonial and heterocolonial victims. This prediction was confirmed, indicating not only that relatedness between the receiver of rescue and the giver of rescue is important [32] but also that community structure is important. Second, the authors performed additional observations with the use of predatory antlion larvae, providing an alternative type of laboratory test of rescue behavior. In the test, the victim ant was dropped into the trap of an antlion kept in a sand-filled cup in the laboratory, and then the potential rescuer was introduced on the flat sand surface near the trap (Figure 3). The authors observed rescue actions with this laboratory setup, and these actions were overall similar to those directed towards the victims ensnared by nylon threads. However, rescue attempts were successful in only 4 out of 58 observations, which seemed a low rate if rescue actions were to be considered an anti-predatory strategy against antlion predation—as more or less directly suggested by some of the works published thus far. Considering that rescue behavior successfulness might have been for various reasons underestimated in laboratory conditions, this low rate still seemed potentially sufficient to be of importance. 

The new type of rescue behavior laboratory test was used in another study, by Miler [38]. In this study, foragers of *F. cinerea* ants differing in life expectancies were dropped into the traps of antlions, and the behavior of nearby nestmates was observed. The life expectancy of the victims was either not manipulated or artificially shortened by CO_2_ poisoning. The author tested the hypothesis that moribund (i.e., poisoned) individuals elicit lower levels of rescue behavior than healthy individuals. The reasons behind this hypothesis were twofold. First, low life expectancy in nature usually means sickness, and sick ants actively avoid interactions [5]; thus, they might avoid eliciting rescue in their nestmates. Ants characterized by low life expectancy might also be simply weaker and so characterized by poorer ability to trigger rescue in their nestmates. Second, the value of individuals to their colonies decreases as their life expectancies decrease [39]; thus, they might be less promptly contacted by their nestmates. The results showed that ants more frequently rescued their nestmates and performed more intensive rescues towards healthy individuals than those with artificially shortened life expectancies. This complemented the study by Nowbahari et al. [33], suggesting that beyond some point, the physiological capability to elicit rescue actions in nestmates probably decreases.

Another study by Nowbahari et al. [40] complicated this picture further. The authors, using laboratory simulations of entrapment, tested the response of *C. cursor* foragers and nurses to entrapped newborn (“callow”) ants. Callow ants in this species were approximately two days younger than previously tested inactives [33]. The authors included tests with homocolonial, heterocolonial and heterospecific callows. The results demonstrated that all callows were rescued quite frequently but that nurses were better able to discriminate between the types of callows. These observations were explained by (1) the lack of a chemical signature in callows, which, when present, allows nestmate recognition in ants, and (2) better experience with brood care and more time spent with callows in nurses than in foragers. Why the ants provided rescue to callows, but did not provide it to only slightly older inactives, as demonstrated in previous research, remained unclear [33,40]. Plausibly, because of the special status of callow (but not inactive) individuals, the former receive increased attention which then translates into higher rescue activity towards them. Indeed, in at least some species of ants, adult individuals initiate prolonged antennal contacts with callows compared to other adults [41].

In a study on the organization of the behavior of *C. cursor* rescuers, Duhoo et al. [42] demonstrated that what ants did during rescue actions in laboratory simulations of entrapment was not a series of random acts. In other words, careful analysis of the behavioral categories of rescue, such as sand digging, sand transport and pulling at the victim, revealed that those categories occurred in sequences. For example, digging was frequently followed by transporting sand, but not the other way around, whereas pulling never occurred after sand transport. These results demonstrated that ants not only display a remarkable ability to target the source of the victim’s endangerment (e.g., bite the snare that is entrapping it) but also show organization in their actions.

Using laboratory tests with *F. cinerea* victims caught by predatory antlions and ensnared by nylon threads, Miler et al. [43] tested whether potential rescuers with differing life expectancies display different levels of rescue behavior. In the previous study on life expectancy, but from the perspective of the victims [38], the two reasons for the effect of life expectancy gave similar predictions; here, the predictions diverged. On the one hand, if ants with a shortened life expectancy actively avoid interactions with nestmates, then they potentially avoid engagement in rescue actions. On the other hand, if individuals with shortened life expectancy have decreased value for their colony, then they might become more prone to sacrifice themselves during risky rescue actions (as in other types of risky tasks, e.g., [44,45]). The authors observed that only in tests with the use of antlions the ants with life expectancy artificially shortened by CO_2_ poisoning engaged less in rescue behavior of their nestmates than the non-manipulated control group. In the other test type, there were no differences between the two groups of potential rescuers. It thus seemed that, generally, shortened life expectancy somewhat increased social withdrawal in these ants, which was well documented in ants in other contexts (e.g., [46,47]). Importantly, however, neither study focusing on the subject of life expectancy [38,43] controlled for changes in other types of behavior, and therefore, the studies did not fully exclude other explanations. For example, the detected effects might have stemmed from general deterioration of activity after CO_2_ poisoning and not an increase in social withdrawal specifically.

The lists of rescuing and non-rescuing species of ants were further expanded by Miler et al. [48]. The authors tested previously unstudied *Camponotus korthalsiae*, *Anoplolepis gracilipes*, *Myrmica ruginodis*, *Iridomyrmex anceps* and *F. polyctena* ants. They additionally re-tested *F. cinerea*. The authors attempted to classify these ants following the hypothetical level of entrapment risk: minimal (*C. korthalsiae*), low (*A. gracilipes*, *M. ruginodis*), moderate (*I. anceps*, *F. polyctena*) and maximal (*F. cinerea*). This categorization was based on two factors, namely, co-occurrence with antlions and likeliness of entrapment situations such as being stuck in clay, under organic debris and/or in plant secretions. The authors, similar to before, used both types of laboratory tests of rescue behavior—with the use of antlions and artificial ensnarement—to additionally gain some insights into the context-dependence of rescue actions in each species. They observed rescue behavior in species classed under “moderate” and “maximal” risk but not the others. This, of course, did not resolve problems detected in earlier research, with some species at apparent risk of entrapment and yet without signs of rescue behavior. There was also an important difference between the test types: *I. anceps* ants showed quite pronounced rescue actions in tests with artificial snares but not in tests with antlions. This demonstrated that, at least in some ant species, the detection of rescue behavior might depend on the test type used for detection. Taken together with the results of another study demonstrating differences between test types [43], these findings showed that the context of rescue action, in terms of the source of endangerment to the victims, matters to potential rescuers.

In a complex study by Frank et al. [49], the authors demonstrated that the evolution of rescue behavior in ants is connected to other factors in addition to various entrapment risks. In the study species *Megaponera analis*, a termite predator, rescue behavior was observed after raids on termite nests. The behavior took the form of picking up and carrying nestmates injured in fights with soldier termites back to the nest to recover. The authors found that 9–15 ants were rescued per day which, considering the low birth rate and colony size in *M. analis* colonies, made up a significant number and explained the evolution of rescue behavior. When the authors experimentally forced already picked up, to-be-rescued individuals to return to the nest alone, 32% of them died, in contrast to 10% of healthy control individuals returning on their own. The main cause of the mortality was predation by spiders on the way back to the nest. Furthermore, the authors found that in this species, blocking stridulation did not affect the probability of being rescued. They covered the stridulation organ of the ants by painting over it with acrylic paint, artificially injured the ants by removing two of their legs, which was a typical type of injury suffered by the ants during fights with termites, and then placed these ants on the track of nestmates returning to the nest after a raid. Such individuals were still rescued, which ruled out stridulation as the source of the rescue behavior releaser signal. Moreover, healthy individuals covered with mandibular gland contents, when placed on the track of nestmates returning to the nest after a raid, were carried back as if they were injured. The authors, using gas chromatography-mass spectrometry, identified substances present in the mandibular gland contents that triggered rescue behavior, namely, dimethyl disulfide and dimethyl trisulfide. Of note, in this context, another study on *F. cinerea* ants by Miler and Kuszewska [50] demonstrated that artificial blocking of the release of mandibular gland contents by gluing ants’ mandibles, as well as covering dummies with the contents of the gland, did not affect rescue behavior expression in laboratory simulations of entrapment. Specifically, in the first case, rescue actions were still observed, whereas in the second case, they were never observed. Quite possibly, in *F. cinerea*, signals triggering rescue behavior originated from anal or venom glands. Naturally, differences between the species in the type of rescue behavior releaser signals are to be expected, but such differences suggest repeated evolution of rescue behavior in ants.

Injured *M. analis* individuals rescued after a raid were found to be treated inside their nest [51], which further expanded knowledge about rescue behavior in this species. The treatment took the form of intense allogrooming. As mentioned before, a typical injury caused to the raiding ants by termites was a loss of extremities. Nestmates were found to lick such wounds on the victims, and when the authors artificially prevented this treatment, the mortality of the injured ants increased from 10% to 80%. Interestingly, the study provided support for the effects of life expectancy indicated by Miler [38] and Miler et al. [43]. Specifically, the authors found that heavily injured ants (i.e., those that lost five extremities), with a lower life expectancy than uninjured or less severely injured individuals, were (1) not rescued on the way back to the nest after a raid and (2) not treated if placed inside the nest. Heavily injured ants had increased social withdrawal—they ignored their nestmates and made it harder to be picked up and carried to the nest.

Two other studies reported yet another context of rescue behavior in ants [52,53]. Desert seed harvester ants *Veromessor pergandei* were found to dismantle spider webs, retrieve ensnared individuals from such webs and groom away the silk that entangled them [52]. The authors found that under natural conditions, nestmates caught in the web significantly enhanced the probability of ants’ removal of that web (i.e., pulling at it, causing it to lose its structure) from the proximity of the foraging trail. Interestingly, 6.3% of individuals engaged in web removal were found to be attacked by the resident spider, demonstrating the riskiness of the behavior. The authors additionally suggested that in this species, similar to *M. analis* [49], rescue behavior releaser signals originated from the mandibular glands. They performed an experiment in which they placed different kinds of toothpicks on a foraging trail of the ants. When the toothpicks had some fresh silk on them or fresh silk wrapped around a dead nestmate, they elicited little reaction. However, when the toothpicks were wrapped with silk that was used to restrain a living nestmate for a few seconds or silk wrapped around a dead nestmate but earlier covered in crushed contents of the head of a freshly killed nestmate, they received marked aggression, similar to real spider webs with a nestmate entangled in them.

In the other mentioned study, similar behavior of nestmate rescue from spider webs was observed in the weaver ant *Oecophylla smaragdina* [53]. The authors investigated experimentally whether rescue occurred towards homocolonial and heterocolonial individuals wrapped in silk—a context in which they observed rescue behavior in the field. Additionally, the authors measured the distance between the focal colony and the foreign colony (i.e., from 2.5 to almost 50 m) tested under heterocolonial conditions. The results demonstrated that rescue always occurred towards nestmates and that the likelihood of rescue was negatively associated with the distance between colonies in the heterocolonial case. Unlike *Tetramorium* sp. E [35], *O. smaragdina* is territorial; therefore, rescue occurrence towards heterocolonial victims was surprising. The authors proposed to explain this result by the familiarity effect—workers from more closely neighboring colonies might encounter each other more frequently and become more tolerant. Alternatively, colonies residing closer might have been more closely related. Interestingly, rescue behavior, whenever it was observed, was always successful (i.e., the victim ant was always freed from the entangling silk).

One of the most recent studies on the topic of rescue behavior in ants addressed the issue of the heritability of rescue activity [54]. The authors noted that in *C. cursor* ants, not all individuals engage in rescue actions towards their nestmates, so some of them are possibly genetically specialized for rescue behavior. In the study, they classified ants as “rescuers” or “non-rescuers”. Rescuers engaged in rescue behavior in response to artificially ensnared nestmate victims during one laboratory simulation of entrapment and then performed the behavior again in another similar simulation. Non-rescuers did not engage in rescue during either test. Then, rescuers and non-rescuers were sacrificed and assigned to patrilines based on molecular methods. Patrilines differentiated these two types of individuals, indicating that rescue behavior in *C. cursor* is at least somewhat heritable. The extent to which individuals in this and other species might be truly dichotomously categorized as “rescuers” or “non-rescuers” is unclear.

The laboratory simulation of entrapment, although initially treated as a simulation of an ant becoming entrapped by collapsing sand or debris [32], at some point came to be viewed as a simulation of an ant falling inside an antlion trap and thus facing a predatory threat as well (e.g., [42,54]). In other words, rescue behavior, particularly in sand-dwelling ants, started to be viewed as evidence of an anti-predatory strategy of the ants, which led to claims of coevolution between ants and antlions [19,20]. The use of the other type of laboratory test, with live antlions, to study rescue actions in ants, probably contributed to this view (e.g., [38]). However, only Taylor et al. [35] attempted to check if rescue actions were successful in this context (as mentioned above, the success rate of rescue actions in antlion traps was low, below 10%, in *Tetramorium* sp. E ants). This issue was readdressed by Turza et al. [55]. The authors measured the success of rescue actions in antlion traps when performed by sand-dwelling *F. cinerea* ants. They found that among 48 tests in which the victim was captured by an antlion with its nestmate present nearby, only in 28 tests (58%) did any signs of rescue behavior occur. Furthermore, none of these rescue actions was successful; in contrast, in 3 of these 28 cases (11%), an already dead victim was released at some point, and the potential rescuer was captured in its place. The authors additionally argued that the timeframe for a successful rescue action in this context is very narrow because antlions inject neurotoxins into their captured prey (e.g., [56]). Thus, the authors urged caution when considering antlion predation as a factor in the evolution of rescue behavior in sand-dwelling ants.

The most recent relevant work considered the fact that the laboratory simulation of entrapment is a versatile test, which can be adapted for different experimental setups [57]. For example, the response to an artificially ensnared victim might be measured in the field, close to the nest entrance (e.g., [34]); in the laboratory, with a group of potential rescuers (e.g., [40]) or with a single potential rescuer (e.g., [50]); in an unmarked area, with a pheromone-free substrate (e.g., [43]); or in a marked area known to the tested ants (e.g., [54]). In addition, it was already indicated by earlier works that the test context might be an important factor in terms of rescue behavior expression [43,48]. Thus, the authors tested *F. cinerea* foragers in five types of scenarios: in the field (1) and the laboratory in an unmarked (2) or marked area (3) with five potential rescuers (4) or a single potential rescuer (5). The results revealed differences, of some general methodological significance, between the tests. First, rescue actions were more probable in the field (occurring in ~80% of tests) than in the laboratory, regardless of the specifics of the latter tests (occurring in ~40–50% of tests). In general, more than one nestmate rarely engaged in rescue during a single test. Rescue actions occurred the quickest in the field, and they lasted the longest there as well. In the laboratory, a marked substrate facilitated rescue expression. Indeed, on such a substrate, rescue actions occurred sooner and lasted longer than those on an unmarked substrate. The primary behavioral categories of rescue observed in the study were pulling at the victims’ legs, digging around the victim, and snare biting. Interestingly, sand transport was extremely rare, despite earlier research showing that it was a quite frequent element of rescue actions in *C. cursor* ants [42]. In general terms, the rescue actions observed with the laboratory type of test including five potential rescuers on a marked substrate seemed to most closely match those in the field. The authors recommended carefully considering the specific type of test to use in future research and noted that in some species tested to date, rescue activity might be underestimated because of the test type that was performed. For example, performing only laboratory tests possibly leads to lowered estimation of rescue activity considering that, in the field, natural environmental cues and proximity to the nest may facilitate risky behavior in ants [57]. 

In another recent study conducted by Santos-Junior et al. [58], the authors modified laboratory simulations of entrapment proposed by Nowbahari et al. [32] even further. In their version of the test, the legs of *Odontomachus brunneus* ants were attached to the floor of the trapping chamber by tape. In the first experiment, they connected the trapping chamber with the arena, in which there were ten potential rescuers. The authors manipulated the distance between the chamber and the arena, placing them 30 cm, 60 cm or 90 cm apart, in each case connecting them by a plastic tract of appropriate length. As a control, they used an empty trapping chamber. In the second experiment, they modified the setup, and the arena with the potential rescuers was connected, by a 25 cm-long tract bifurcated at the end, to two trapping chambers containing (1) a free nestmate and an immobilized nestmate, (2) an immobilized nestmate and a heterocolonial immobilized individual, or (3) an immobilized nestmate and a heterospecific immobilized individual. In the first experiment, the ants entered the trapping chamber with a nestmate more often than the control chamber without a nestmate, but only from 30 cm away. In the second experiment, on the other hand, the ants opted for the trapping chamber with an immobilized nestmate over that with a loose nestmate and a heterospecific immobilized individual but not a heterocolonial immobilized individual. Rescue behaviors took the form of biting, pulling and/or stinging the tape. The authors argued that the trapped ants release relatively short-range “call for help” signals that, in the case of *O. brunneus*, may be species-specific chemical and/or stridulatory signals. Rescue actions towards heterocolonial victims were reported in earlier works [35,53], and the authors argued that in all cases, this might stem from a failure of recognition on the part of the potential rescuer(s).

## 3. Rescue Behavior in Other Taxa

Cases of rescue behavior are also known in taxa other than ants. These taxa include numerous primate species [59,60,61,62,63,64,65,66,67], other mammals [68,69,70,71,72,73] and, to date, a single bird species [74]. The occurrence of rescue actions in such a diverse group of taxa speaks to the generality of the phenomenon and its prevalent importance. All of these taxa are group-living and their rescue activity is most often connected to helping group members escape predation or aggression. In the context of rescue behavior, rats are by far the most studied group of animals other than ants. Below, we briefly introduce studies devoted to rescue behavior in rats, primarily in order to illustrate some questions thus far not addressed in ants at all.

In a study by Ben-Ami Bartal et al. [75], a laboratory setup similar to that introduced for ants by Nowbahari et al. [32] was used. In this setup, one rat, the victim, was placed inside a restrainer in an arena. Another individual, its familiar cagemate, the potential rescuer, was then introduced into the arena. The potential rescuers circled the restrainer when it trapped the victim rat and showed behavior that was strikingly similar to that of the rescuing ants, i.e., digging at and biting the restrainer. After some time, they also learned how to open the restrainer to release the victim, which they did frequently. The authors conducted an experiment, in which they observed the behavior of the potential rescuers towards two restrainers placed in the arena: one containing the victim and another containing chocolate or one empty and another containing chocolate. For rats, chocolate is highly palatable. The authors found that the time to open the restrainer was lower for that containing chocolate when the other restrainer was empty but not when the other restrainer held a cagemate. Overall, the authors argued that the rats acted to help the victims, even at a cost of something tasty to eat, and that this stemmed from their empathy—goal-directed behavior of improving another’s wellbeing. This “empathy-based” interpretation of the results was later criticized theoretically [76] and experimentally [77]. Of particular importance, Silberberg et al. [77] argued that the results were better explained by the pursuit of social contact than by empathy. In other words, the rats, which are highly social, might have released the victims to interact with them.

In another study [78], the authors investigated the effect of previous social experience on rescue behavior expression. They found, among other things, that individuals raised by rats from another strain provided no help to rats of their own strain as adults, rescuing only individuals of the foster strain. The authors argued that in rats, group identification is key to rescue expression. Shortly later, a new type of test for rescue behavior in rats was introduced [79]. The authors tested rats in chambers connected by sliding doors. In one of the experiments, there were two chambers, one with a water pool and another with solid ground. The victim was dropped into the pool, and the potential rescuer, released into the other chamber, had to slide the door open to help the victim escape the aversive stimulation of being in the water. Rats frequently opened the door. Additionally, the authors demonstrated in a role-reversal test that if the rats switched places and the victim in one test became the potential rescuer in another test then the time to open the door was shorter for a rescuer with previous experience as a victim. In other words, rats experiencing aversive stimulation from which they later had to rescue the victim proved to be better (i.e., quicker) rescuers than naïve individuals. In this study, empathy was yet again invoked as the explanation for the behavior of rescuing rats, and this interpretation was again experimentally criticized, this time by Schwartz et al. [80]. Indeed, the authors argued that it was again the pursuit of social contact that better explained the results.

Ben-Ami Bartal et al. [81] again argued that they provided evidence in favor of empathy-driven rescue behavior in rats. The authors used their established setup with the restrainers. In the study, the potential rescuers treated with an anxiolytic drug displayed reduced helping activity. That drug supposedly blocked “affective processing”, so the authors concluded that affect (i.e., empathic concern) was necessary to motivate rescue behavior. Interestingly, a similar result was obtained with heroin [82]. At that point, the empathy-based explanation of rescue activities in rats was again attacked [83,84]. Indeed, Hachiga et al. [83] studied rats in an E maze, where the animals needed to choose between entering (1) an empty chamber or a chamber with a restrained rat, (2) a chamber with a restrained rat or a chamber with an unrestrained rat and (3) an empty chamber or a chamber with an unrestrained rat. This design was used to differentiate between two competing explanations for the apparent rescue actions in rats—empathy or pursuit of social contact—particularly in condition (2). The results provided evidence against empathy-based behavior, as the rats did not discriminate between restrained or unrestrained cagemates in condition (2). In other words, the results demonstrated that rats probably engage in rescue because they seek interaction. In turn, Blystad et al. [84] studied the time to open a restrainer when it held some food, held a cagemate or was empty. The results clearly showed that the time was shortest for food, and the authors, therefore, argued that food takes priority over social interaction in food-deprived rats.

Yamagashi et al. [85] introduced a modified test type with the use of an apparatus with a pool chamber and solid ground chamber connected by sliding doors [79]. The authors demonstrated that rats provided help to the victim rats even when they were total strangers. In one of the most recent studies [86], the authors aimed to, first and foremost, eliminate the possibility that rats rescue their cagemates because they seek social contact with rescued individuals. In a three-chamber empathy apparatus, they had the victim in the water chamber, the potential rescuer in the dry chamber, and another dry chamber into which the victim was released when the rescuer pulled the chain opening the door leading out from the water chamber. The results demonstrated that rats rescued the victim when they had no opportunity to meet it—because it was released into another, separated chamber. Thus, the authors claimed that their study showed the irrelevance of social rewards for rescue occurrence. These results were then followed by another study [87]. Perhaps most importantly, the authors showed that rats that previously rescued their cagemates from the restrainers showed no decrease in the frequency of opening or the time to open the restrainers when they were then presented to them empty. In other words, the action of opening the restrainers persisted in rats, even in the absence of a cagemate. The authors suggested the possibility that the rats might open the restrainers with a cagemate inside to take its place, i.e., to shelter themselves from the aversive open test arena. Then, Heslin et al. [88] demonstrated no preference for helping when rats were offered an opportunity to release a restrained victim, engage a free rat, or not socialize.

In the study by Havlic et al. [89], the authors investigated the so-called bystander effect. This well-known human phenomenon describes a situation in which a person in the presence of passive people, when someone requires help, shows a decreased probability of giving help. As demonstrated by the authors, rats exposed to victims (i.e., restrained individuals) in the presence of potential rescuers rendered incompetent by pharmacological treatment delivered fewer consecutive openings of the restrainers. In turn, the presence of competent potential rescuers resulted in an increase in helping. Overall, the study demonstrated high conformity in terms of rats’ helping behavior. 

Whether empathy or other processes—such as the pursuit of social contact—drive the expression of rescue behavior in rats is obviously a central issue of debate in the abovementioned studies conducted on rats. It is of limited interest in the context of studies on ants. All of the interpretations in studies conducted on ants look for drivers of rescue behavior elsewhere. Research on rats, however, highlights some areas in which studying ants and their rescue behavior might be especially useful. We return to this in the next section of the review.

## 4. Identifying Research Gaps

The ant studies described above may be roughly categorized as devoted to shedding some light on (1) variation in rescue behavior activity on a between-individual scale, (2) factors contributing to the evolution of rescue behavior on a between-species scale, (3) rescue behavior releaser signals and (4) rescue behavior benefits and costs. In each category, obvious research gaps might be indicated to guide further research. Moreover, additional interesting research questions might be identified based on studies of rats (5).

### 4.1. Variation in Rescue Behavior Activity on a Between-Individual Scale

In the first category, although some work has already been done on the influence of the division of labor and community structure, it needs to be pointed out that ants show enormous diversity in these terms. Studying rescue expression in ants characterized by various forms of division of labor and community structure might bring us closer to a better understanding of the patterns of rescue activity in different individuals. In terms of the division of labor, for example, numerous species demonstrate elaborate polyphenisms, with not only reproductive queens, males and sterile workers but also, for instance, soldiers, minor workers and major workers, as well as extreme size variation in workers of apparently the same sub-caste (e.g., [90,91]). In terms of community structure, on the other hand, a very interesting and unstudied issue is presented by socially parasitic ants, including the so-called “guest ants”, “temporary social parasites” and “slave-making ants”, which are to a lesser or greater extent dependent on their host ant species. Guest ants use the host nest structures and the hosts themselves, escaping their aggression using chemical mimicry and the production of host-like odors [92]. Temporary social parasites use host colonies to initiate new nests, which leads to the creation of temporary mixed colonies of parasites and hosts [93]. Slave-making ants (see [27]) rob host ants’ nests and rear captured individuals in their own nests. Interestingly, slaves reared in colonies of slave-making parasites do not recognize their status as slaves [94]. There are some 230 species of such parasitic ants worldwide [93], presenting a unique opportunity to test whether rescue behavior might, under special conditions, occur even towards completely unrelated heterospecifics. Other than these two issues, there might be various factors mediating whether individuals demonstrate rescue behavior, such as the health status of workers, their individual experience in terms of rescue activity or the level of relatedness within the colony—all waiting to be explored experimentally in more detail. Of particular interest are also unstudied issues of individual consistency in engaging in rescue activity as well as those of ant personalities and whether being a “rescuer” correlates with other behaviors, forming larger behavioral phenotypes.

### 4.2. Factors Contributing to the Evolution of Rescue Behavior on a Between-Species Scale

In the second category, it is worth noting that only a few species of ants have been tested for rescue behavior occurrence to date (Table 1). Only by investigating new species might some general patterns become evident and elucidate why rescue actions occur in some but not all species and, of particular importance, why some species in hypothetical need for rescue show no rescue behavior. Importantly, researchers should never neglect to provide information about species displaying no rescue behavior. It might be especially insightful to study species closely related to those already studied, in the hope of determining the importance of phylogeny for rescue activity. It is clear that rescue behavior occurs in distantly related ant species (Table 1), which makes it likely that it evolved multiple times, but its prevalence in related species is currently difficult to assess. Moreover, rescue actions have been reported for only three ecological contexts, namely, hunting injury [49,51], spider predation [52,53] and, more anecdotally, burial under the substrate or debris. In terms of antlion predation and rescue actions as an antipredatory strategy of ants in response to selective pressures imposed on them by antlions, this has not been satisfactorily demonstrated to date, and the fact that some ants display rescue in this context might even be an artefact. Curiously, the last-mentioned study on ants, by Santos-Junior et al. [58], provided another indirect clue in favor of this argument. Specifically, *O. brunneus* ants, in which the authors demonstrated rescue of nestmates trapped by tape, nevertheless show an alternative tactic when faced with antlion predation, namely, escape jumps from the traps [95]. Therefore, in general, tactics other than rescue for escaping antlion predation in ants, such as avoidance of high-density antlion aggregations [96], might be more important. Overall, further documentation of ecologically relevant contexts in which ants show rescue actions is needed to navigate the more than 16,000 described species of ants [97] in search of general patterns of rescue presence or absence. 

### 4.3. Rescue Behavior Releaser Signals

In the third category, many more mechanistic studies directly identifying the signals responsible for rescue behavior elicitation are needed. This might help reveal general patterns and help predict evolutionary lineages with more and less probable rescue actions and in such a way inform and complement investigations devoted to the evolution of rescue behavior on a between-species scale. In more general terms, very little focus has been devoted to the behavior of the victim (i.e., the individual in need of rescue). Currently, the image of that individual is rather passive—it finds itself in need of rescue and signals this need—but what else is it doing? Only Frank et al. [49] noted that rescue in *M. analis* ants is impossible without the cooperation of the individual in need of rescue (i.e., it must assume an appropriate position to be picked up by a nestmate and carried to the nest). Other than that, early life stages (i.e., egg, larva, and pupa) have not yet been investigated in terms of their rescue elicitation abilities and, if rescue actions towards early-life stage individuals occur, whether such rescue actions rely on the same mechanisms as in the case of adults. As suggested in one of the studies by Nowbahari et al. [40], young individuals might receive special treatment within the colony when it comes to rescue. Curiously, in many host species invaded by the already mentioned slave-making ants, some individuals evacuate the larvae during a raid of the slave-makers [94]. This behavior seems worth investigating in the context of the parallels it presents with potential rescue actions directed towards adults in these species.

### 4.4. Rescue Behavior Benefits and Costs

In the fourth category, the balance of costs and benefits of engagement in rescue behavior surely needs extensive research attention. Except for a few observations of the death of an individual performing rescue action [27,35,52], the costs of rescue behavior are more often assumed than explored experimentally (but see [49] and [52] for calculations of wasted opportunities under no rescue directed towards individuals in need of help). These assumed costs of rescue involve, for instance, time spent otherwise on tasks such as food collection. The value of individuals—from the perspective of the whole colony—surely differs both among individuals of a single colony and as an average characteristic between species and thus might provide some explanation for variation in rescue occurrence within and among species. In this context, the dependence of rescue behavior on the number of individuals in the colony requires attention as well. Ants show an unimaginably large range in terms of the maximum and minimum numbers of individuals in a single colony. Colony size depends on colony structure (e.g., monodomy, polidomy or a supercolony), which is characteristic of a given species [98], as well as the age of the colony and access to food resources, all of which might affect rescue activity. Moreover, what is conspicuously missing from research on rescue behavior in ants to date is the whole-colony perspective. For instance, it might be insightful to ask whether colonies differing in their average rescue activity differ in fitness outcomes when forced to cope with ecologically relevant situations that elicit rescue behavior (e.g., nest collapse). Such research might provide support for the notion that, in ants, a colony is a higher-order unit of selection than the individual [4,98].

### 4.5. Further Research Gaps Identified Based on Studies of Rats

Ants and rats show strikingly similar behaviors, which opens up a rich possibility for conducting unique comparative analyses that might be informative to researchers working on both taxa. The pursuit of social contact in ants and its role in the expression of their rescue behavior, for example, seem especially worth investigating. Manipulating social contact deprivation is fairly easy in ants and might involve both developmental effects, with individuals raised in different social environments, and more immediate effects, with individuals manipulated in terms of the size of their social network or the frequency of their interactions with nestmates. Such research, devoted to the effect of social deprivation on rescue behavior expression, might prove discerning. Finding the effect of social isolation on rescue behavior is likely as other types of behavior, such as nestmate recognition or trophallaxis, change in ants following a period of social isolation [99,100].

Studies conducted on rats reveal how many interesting issues remain completely unaddressed in ants. These issues include the priority effects, or the study of ants forced to choose between helping a nestmate and performing some other behavior. The alternative behavior might take the form of, for instance, food collection. An additional, potentially important factor accounted for in such an experimental setup might include the level of food deprivation in the potential rescuer. Another issue concerns the role reversal effects, with ants switching between providing and receiving help. This might put some light on the importance of individual experience in terms of rescue performance. Moreover, the drug and/or hormone effects might also be relatively easily studied in ants, by manipulating their levels of, for example, biogenic amines. Potentially, the expression of rescue behavior in ants might be even modified and, thus, controlled, by their diet. Additionally, the bystander effects, in the form of other individuals and their influence on the expression of rescue in known rescuers, awaits investigation. These issues, when ultimately studied, need to be discussed in the context of research on rats. Notably, there already are some curious areas of overlap between studies conducted on ants and rats (e.g., group familiarity and identification, [53,78]), indicating common areas of interest and potential for elaboration. Naturally, secondary use of experimental designs applied for rats to study ants and plain replication is of rather limited value. Nevertheless, novel comparative studies might inform research on rescue behavior on an unprecedented scale.

## 5. Conclusions

The study of rescue behavior in ants is largely incomplete. Here, we have tried to provide an up-to-date summary of research on the subject and, thus, to identify the most pressing gaps in the knowledge, particularly in relation to earlier works on ants and other taxa. There is still much to learn about the ecology and evolution of rescue behavior and—in a broader context—cooperation. The stark contrast between the ongoing discussions of research on ants and rats and their rescue behaviors represents a well-known division in how helping is viewed in research on “lower” and “higher” animals [101,102], but it does not mean that integration of concepts is impossible. Our synthesis will, hopefully, inspire novel research in the areas of special interest that we highlighted here. No less importantly, we also await reports of rescue actions in other, new taxa, which might elucidate more general patterns of altruism occurrence among animals. 

## Figures and Tables

**Table 1:** List of ant species studied for their rescue activity. Asterisks (*) mark species with potentially somewhat underestimated activity (as suggested in [57]) in terms of, for example, frequency or behavioral categories of rescue.

Species	Testing Procedure	Rescue	Reference
*Anoplolepis gracilipes*	artificial ensnarement in the laboratory antlion traps in the laboratory	undetected *	[48]
*Aphaenogaster senilis*	artificial ensnarement in the field	detected but weak and/or infrequent	[34]
*Atta cephalotes*	entrapment using soil	detected and pronounced *	[24]
*Camponotus korthalsiae*	artificial ensnarement in the laboratory antlion traps in the laboratory	undetected *	[48]
*Cataglyphis floricola*	artificial ensnarement in the field	detected and pronounced	[34]
*Cataglyphis cursor*	artificial ensnarement in the field and in the laboratory	detected and pronounced	[32,33,40,42,54]
*Formica cinerea*	artificial ensnarement in the field and in the laboratory antlion traps in the field and in the laboratory	detected and pronounced	[27,38,43,48,50,55,57]
*Formica fusca*	antlion traps in the field	undetected	[27]
*Formica polyctena*	artificial ensnarement in the laboratory	detected but weak and/or infrequent *	[48]
*Formica sanguinea*	antlion traps in the field	detected and pronounced	[27]
*Iridomyrmex anceps*	artificial ensnarement in the laboratory	detected but weak and/or infrequent *	[48]
*Megaponera* *analis*	confrontation with termites in the field	detected and pronounced	[49,51]
*Messor barbarus*	artificial ensnarement in the field	undetected	[34]
*Messor marocanus*	artificial ensnarement in the field	detected but weak and/or infrequent	[34]
*Myrmica ruginodis*	artificial ensnarement in the laboratory	undetected *	[48]
*Lasius grandis*	artificial ensnarement in the field	detected and pronounced	[34]
*Pogonomyrmex occidentalis*	entrapment using sand	detected and pronounced *	[25]
*Prenolepis imparis*	artificial ensnarement in the field antlion traps in the laboratory	detected but weak and/or infrequent	[35]
*Oecophylla smaragdina*	spider webs in the field	detected and pronounced	[53]
*Odontomachus brunneus*	artificial ensnarement in the laboratory	detected but limited to aggression towards the tape *	[58]
*Solenopsis geminata*	artificial entrapment in the laboratory	detected and pronounced *	[26]
*Tetramorium* sp. E	artificial ensnarement in the field antlion traps in the laboratory	detected and pronounced	[35]
*Veromessor pergandei*	spider webs in the field and in the laboratory	detected and pronounced	[52]

## Data Availability

Not applicable.
